# Factors associated with hypertriglyceridemia among the hill tribe people aged 30 years and over, Thailand: a cross-sectional study

**DOI:** 10.1186/s12889-021-10632-z

**Published:** 2021-03-23

**Authors:** Panupong Upala, Tawatchai Apidechkul, Chanyanut Wongfu, Siriyaporn Khunthason, Niwed Kullawong, Vivat Keawdounglek, Chalitar Chomchoei, Fartima Yeemard, Ratipark Tamornpark

**Affiliations:** 1 Center of Excellence for The Hill tribe Health Research, Mae Fah Luang University, Chiang Rai, Thailand; 2 School of Health Science, Mae Fah Luang University, Chiang Rai, Thailand; 3 Chulabhorn Royal Academy, Bangkok, Thailand

**Keywords:** Hypertriglyceridemia, Hill tribe, Prevalence, Factor associated

## Abstract

**Background:**

Triglycerides are lipids in the human body that are produced from the consumption of daily food and drink. However, elevated serum triglycerides, also known as hypertriglyceridemia (HTG), are key biomarkers indicating an unhealthy status and increased risks of cardiovascular diseases (CVDs) and pancreatitis. Different groups of people have different patterns and styles of cooking and different patterns of consumption, such as hill tribe people, who have their own unique culture and cooking practices. This study aimed to estimate the prevalence of and determine the factors associated with HTG among the hill tribe population in Thailand.

**Method:**

A cross-sectional study was performed. Data and a-5 mL blood sample were collected from participants who were members of one of the six main hill tribes in Thailand: Akah, Lahu, Hmong, Yao, Karen, and Lisu. People who lived in 30 selected hill tribe villages and aged 30 years over were asked to participate the study. Pearson correlation and logistic regression were used to detect the correlations and determine the associations between variables, respectively, at a significant level of α = 0.05.

**Results:**

A total of 2552 participants participated this study; 65.9% were females, 72.35% were aged 40–69 years, 76.7% had no education, 48.7% worked in the agricultural section, and 71.2% had an annual income of less than 50,000 baht/family. Regarding the triglyceride level, 41.7% of participants had elevated levels of serum triglyceride or HTG; 16.4% had a borderline high level, and 25.3% had a high level. After controlling for all potential confounder factors, three variables were found to be associated with elevated serum triglycerides. Those who were members of the Lahu and Hmong tribes were 1.62 times (95%CI = 1.25–2.01) and 1.63 times (95%CI = 1.23–2.16) more likely to have elevated serum triglycerides than those who were members of the Akha tribe, respectively. Those who used a high quantity of cooking oil for daily cooking were 0.73 times less likely to have an abnormal level of triglycerides than those who used a low quantity of cooking oil for daily cooking (95%CI = 0.58–0.91), and those who had a waist circumference indicating obesity were 1.28 times more likely to have an abnormal level of triglycerides than those who had a normal waist circumference (95%CI = 1.08–1.52).

**Conclusion:**

Public health programs that focus on encouraging people to have regular exercise to reduce their body weight, particularly in some tribes, such as Lahu and Hmong, should be implemented.

**Supplementary Information:**

The online version contains supplementary material available at 10.1186/s12889-021-10632-z.

## Background

Triglycerides are a type of blood lipid that, at healthy levels, can help maintain health and support many functions in the human body [1]. However, when triglyceride levels become too high, they may have negative impacts on health [2, 3]. High triglycerides have been found to be major contributors for many severe diseases that may require chronic care or even lead to death, including cardiovascular diseases (CVD) [4–6], stroke [7], pancreatitis [8], atherosclerosis [9], etc. Several factors have been demonstrated to serve as risk factors for hypertriglyceridemia (HTG), including overweight or obesity [4]. In 2016, the World Health Organization (WHO) reported that globally, 1.9 million adults and 650 million adults were overweight and obese, respectively [10]. Several other lifestyle and health behaviors have also been indicated as risk factors for HTG in a population. In 2019, the prevalence of HTG was reported at 10.0% among the adult population in several countries in Europe [11], while 25.1–33.3% were reported among the adult population of the United States [12]. The Thailand National Health Survey which was conducted in 2014, the prevalence of HTG was reported at 38.6% among Thai adult population [13].

Thailand has almost 67 million people living in four different regions: northern, southern, northeastern, and central regions [14]. The people of each region have different styles of cooking, follow different food preparation practices and have different flavor preferences [15]. Many of the foods and cooking practices used differ based on the socioeconomic factors of a country, particularly among poor people who have few choices [16]. This culinary inequality is a common characteristic in all developing countries that are facing the large problem of unequal economic development. People who live in a big city have a better chance of obtaining a good job and a good income compared with those who live in the countryside or in rural settings or those who mainly work in the agricultural sector, which is classified as a country’s lowest income group [17]. The northern region is known as the second poorest region of Thailand, and a large proportion of the people residing in this region are part of ethnic minority groups [18]. Hill tribe people are considered one such ethnic minority group, and they often live below the national poverty level.

The hill tribe is a group of people who have migrated from the south of China to northern Thailand over the past few centuries [19]. These people are classified into six main groups: Akha, Lahu, Hmong, Yao, Karen, and Lisu [19]. Each tribe has its own culture and lifestyle, which are particularly distinct in its daily cooking practices and preferred flavors [20]. Historically, hill tribe people used animal oils as their main cooking oil [21], but due to conditions of economic constraint and transportation challenges, many kinds of vegetable oils are now easily available, even in small villages located far from any big city [22]. However, few hill tribe people in Thailand are educated under the Thai educational system, and health information or key health messages produced in the Thai language are therefore often not accessible to these populations [23]. With several conditions of the hill tribe people in Thailand including the barriers of language and distance of their residence, there is no scientific information relevant to hypertriglyceridemia available especially the risk factors of hypertriglyceridemia.

Currently, there is little information exists regarding abnormal lipid profiles and, particularly, HTG, which could lead to severe health problems, as mentioned earlier, within the hill tribes. Therefore, this study aimed to estimate the prevalence of and determine the factors associated with HTG among hill tribe people in Thailand.

## Methods

### Study design and participants

A cross-sectional study was applied to collect the information from the participants. The study population was composed of individuals 30 years of age and older who belong to one of the six main hill tribes: Akha, Lau, Hmong, Yao, Karen, and Lisu. In 2019, there were 749 hill tribe villages in Chiang Rai, which included 316 Lahu villages, 243 Akha villages, 63 Yao villages, 56 Hmong villages, 36 Karen villages, and 35 Lisu villages [24]. Five villages in each tribe were randomly selected by the computer generated simple random method from the lists of the hill tribe villages located in Chiang Rai Province, Thailand [24]. Individuals from 30 selected villages were eligible to participate in the study. However, those who could not provide all of the essential information related to the study protocols or who were not able to provide a blood sample on the day of data collection were excluded from the study.

### Sample size estimation

The sample size was calculated based on the standard formula for a cross-sectional design: n = [Z^2^_α/2_PQ]/ e^2^, where Z^2^_α/2_ = 1.96, *P* = 0.39 [13], Q = 0.61, e = 0.05, then 366 participants were required from each tribe. Therefore, at least 2196 participants were needed for the analysis. After adding 15.0% for any error during the study, a minimum of 2525 participants were needed in the whole study.

### Research tools

A validated questionnaire and 5 mL blood specimens were used as the research tools. The questions were developed by researchers based on a literature review and included the information obtained from in-depth interviews with four health care professionals and five village health volunteers who were working in the different hill tribe villages. The questionnaire’s content was validated using the item objective congruence method by three experts in the field, including one public health professional with significant experience in the field, one public health nutritionist, and one epidemiologist. Each item had three choices: − 1 signified that it was not related to the content of the study, 0 meant that it required revision before use, and + 1 signified that the question completely reflected the content of the study. The scores from the three experts were pooled and recalculated to form the overall interpretation. If the total score was less than 0.5, the question was removed from the questionnaire. If the question scored 0.5–0.7, it was revised based on the experts’ comments, and if the total score was greater than 0.7, it was included as-is in the questionnaire. Next, the questionnaire was piloted among 20 individuals who had characteristics similar to those in the study populations. The main objective for the pilot test was to determine the feasibility of use of the questions with the hill tribe populations, including any concerns related to any of the questions violating their cultural beliefs. The test was also extended to examine the order of the questions and the participants’ comprehension of the questions. Finally, the questionnaire consisted of four parts. Part one included 9 questions that were used for collecting participants’ general information, such as age, sex, religion, education level, etc. Part two included 10 questions to assess risk behaviors, such as smoking, alcohol use, amphetamine use, or opium use, etc. The questions on asking substances use, which intended to ask whether they had experience or current use of the substance, two options were provided for the response in each question; “yes” or “no”. Part three included 5 standard questions (ST-5) [25] used to assess participants’ stress and 9 standard questions (PHQ-9) [26] to assess for depression. The final portion included 7 open questions to document the physical examination findings (weight, height, blood pressure) and laboratory results (lipid profiles and HbA1c) (Additional file 1, Questionnaire).

### Independent variables

Body weight index (BMI) is also a standard used by the WHO that is classified into three categories: underweight (≤18.49), normal weight (18.50–24.99), and overweight (≥25.00) [27]. Waist circumference was classified as either normal (< 90 cm) or elevated (≥90 cm) for males, and either normal (< 80 cm) or elevated (≥80 cm) for females [28]. Stress was classified into three categories [25]; low (≤4 scores), moderate (5–7 scores), and high (≥8 scores). Depression was classified into two categories [26]; no (≤6 scores) and yes (≥7 scores).

### Outcome variable

The triglycerides were classified into two major groups according to guidelines from the World Health Organization [29]: normal (< 150 mg/dL) and abnormal (≥150 mg/dL). Abnormal levels were further classified into borderline high (150–199 mg/dL), high (200–499 mg/dL), and very high (≥500 mg dL). In this study, those who were diagnosed and treated as HTG patients with or without abnormal triglycerides were included into a HTG group.

### Data gathering procedure

Access to the villages was granted by the district government officers. Village headmen were contacted and provided brief information regarding the study, including a date for a future meeting. During the meeting with the village headman, all essential information related to the study was provided, and the researchers asked to obtain a list of village members who met the study criteria. Researchers returned to the villages again after receiving the list of villagers who met the study criteria. An appointment was made 3 days in advance of data collection, including requesting participants fasted (nothing per oral (NPO)) for 12 h. prior to blood specimen collection. On the day of data collection, all participants who met the study criteria were provided the complete information related to the study in both Thai and the local language with the help of village health volunteers fluent in both Thai and the local languages. After verifying that they met the criteria, the participants were asked to complete the informed consent form before a 5 mL blood specimen was collect, followed by a 15-min interview to complete the questionnaire. Blood specimens were properly stored and transferred to the Mae Fah Laung Medical Laboratory Center for laboratory testing on the same day.

### Data analysis

Questionnaires were checked for completion and coded before double entry into Excel spreadsheets, and then the data file was transferred into the SPS program (version 24, Chicago, IL). Proper descriptive statistics were used for the participants’ characteristics. The mean and SD were included for continuous data with normal distribution, while the median and IQR were used for those data that did not follow a normal distribution. Percentages were used for nominal and ordinal data. The Pearson correlation was used to assess the correlations between the continuous variables, such as age, SBP, DBP, HDL-C, LDL-C, and Hb1Ac. Logistic regression was used to detect factors associated with triglyceride abnormalities. The mode of “ENTER” was used to select the variable into the model in both univariate and multivariate analyses according to the conceptual framework of the study at the significant level of α = 0.05. The fitting of the model was guided by the pseudo R^2^ and the Hosmer-Lemeshow chi-square. In the multivariate step, some variables were controlled before making the interpretation at the significant level of α = 0.05.

## Results

A total of 2552 participants were recruited into the study; 65.9% were female, 72.35% were aged 40–69 years (mean = 54.1, SD = 13.1), and 79.9% were married. The majority had received no education (76.7%), Buddhist (54.2%), worked in agricultural section (48.7%), and had an annual income less than 50,000 baht/family with the median at 30,000, and interquartile range = 45,000 (71.2%). One-fifth had stress at a moderate to high level (18.6%), and 12.0% had depression. A large proportion of participants did not exercise (60.8%), 24.3% smoked, and 23.4% used alcohol (Table 1).

**Table 1:** Factors associated with triglyceride among the hill tribe aged 30 years and over in univariate and multivariate analyses

Factors	Total n (%)	Triglyceride		OR	95%CI	***p***-value	AOR	95%CI	***p***-value
		Abnormal n (%)	Normal n (%)						
**Total**	2552 (100.0)	1064 (41.7)	1488 (58.3)	N/A	N/A	N/A			
**Sex**									
Male	870 (34.1)	355 (33.4)	515 (34.6)	1.00					
Female	1682 (65.9)	709 (66.6)	973 (65.4)	1.06	0.90–1.25	0.513			
**Age** (years)									
30–39	368 (14.4)	153 (14.4)	215 (14.4)	1.00					
40–49	619 (24.3)	279 (26.2)	340 (22.8)	1.15	0.89–1.50	0.284			
50–59	649 (25.4)	260 (24.4)	389 (26.1)	0.94	0.72–1.22	0.637			
60–69	578 (22.6)	229 (21.5)	349 (23.5)	0.92	0.71–1.20	0.550			
70–79	271 (10.6)	116 (10.9)	155 (10.4)	1.05	0.77–1.45	0.756			
≥ 80	67 (2.6)	27 (2.5)	40 (2.7)	0.95	0.56–1.61	0.845			
**Tribe**									
Akha	714 (28.0)	289 (27.2)	425 (28.6)	1.00			1.00		
Lahu	391 (15.3)	199 (18.7)	192 (12.9)	1.52	1.19–1.95	0.001*	1.62	1.25–2.01	< 0.001**
Hmong	389 (15.2)	195 (18.3)	194 (13.0)	1.48	1.15–1.90	0.002*	1.63	1.23–2.16	0.001**
Yao	368 (14.4)	130 (12.2)	238 (16.0)	0.83	0.62–1.04	0.100	0.89	0.67–1.20	0.455
Karen	408 (16.0)	131 (12.3)	277 (18.6)	0.70	0.54–0.90	0.005*	0.81	0.60–1.08	0.154
Lisu	282 (11.1)	120 (11.3)	162 (10.9)	1.09	0.82–1.44	0.548	1.20	0.88–1.63	0.243
**Religion**									
Buddhist	1383 (54.2)	554 (52.1)	829 (55.7)	1.00					
Christ and Muslim	1169 (45.8)	510 (47.9)	659 (44.3)	1.16	1.09–1.36	0.049*			
**Education**									
No-education	1957 (76.7)	840 (78.9)	1117 (75.1)	1.00					
Primary school	365 (14.3)	140 (13.2)	225 (15.1)	0.83	0.66–1.04	0.105			
Secondary school	189 (7.4)	75 (7.0)	114 (7.7)	0.88	0.65–1.12	0.390			
High school and higher	41 (1.6)	9 (0.8)	32 (2.2)	0.37	0.18–0.79	0.010*			
**Occupation**									
Unemployed	628 (24.6)	252 (23.7)	376 (25.3)	0.93	0.74–1.15	0.492			
Agriculturist	1243 (48.7)	526 (49.4)	717 (48.2)	1.01	0.84–1.22	0.892			
Other	681 (26.7)	286 (26.9)	395 (26.5)	1.00					
**Annual income** (baht)									
≤ 50,000	1816 (71.2)	769 (72.3)	1047 (70.4)	1.00					
50,001-100,000	527 (20.7)	211 (19.8)	316 (21.2)	0.91	0.75–1.12	0.344			
≥ 100,001	209 (8.2)	84 (7.9)	125 (8.4)	0.92	0.68–1.23	0.550			
**Marital status**									
Single	138 (5.4)	63 (5.9)	75 (5.0)	1.00					
Married	2039 (79.9)	856 (80.5)	1183 (79.5)	0.86	0.61–1.22	0.398			
Other	375 (14.7)	145 (13.6)	230 (15.5)	0.75	0.51–1.11	0.154			
**Stress** (ST-5)									
Low	2076 (81.3)	867 (81.5)	1209 (81.3)	1.00					
Moderate	399 (15.6)	165 (15.5)	234 (15.7)	0.98	0.79–1.22	0.879			
High	77 (3.0)	32 (3.0)	45 (3.0)	0.99	0.63–1.57	0.971			
**Depression**									
No	2245 (88.0)	937 (88.1)	1308 (87.9)	1.00					
Yes	307 (12.0)	127 (11.9)	180 (12.1)	1.01	0.77–1.30	0.902			
**Exercise**									
No	1551 (60.8)	644 (60.5)	907 (61.0)	0.83	0.64–1.07	0.153			
Sometimes	719 (28.2)	290 (27.3)	429 (28.8)	0.79	0.60–1.04	0.097			
Regularly	282 (11.1)	130 (12.2)	152 (10.2)	1.00					
**Smoking**									
No	1932 (75.7)	812 (76.3)	1120 (75.3)	1.00					
Yes	620 (24.3)	252 (23.7)	368 (24.7)	0.95	0.79–1.14	0.543			
**Alcohol drinking**									
No	1955 (76.6)	820 (71.1)	1135 (76.3)	1.00					
Yes	597 (23.4)	244 (22.9)	353 (23.7)	0.96	0.79–1.15	0.642			
**Amount of salt use for cooking**									
High	1193 (46.7)	474 (44.5)	719 (48.3)	1.20					
Moderate	1115 (43.7)	478 (44.9)	637 (42.8)	0.78	0.59–1.03	0.074			
Low	244 (9.6)	112 (10.5)	132 (8.9)	0.88	0.67–1.17	0.387			
**Amount of monosodium glutamate use for cooking**									
High	1298 (50.9)	521 (49.0)	777 (52.2)	0.83	0.63–1.09	0.176			
Moderate	1006 (39.4)	432 (40.6)	574 (38.6)	0.93	0.70–1.23	0.605			
Low	248 (9.7)	111 (10.4)	137 (9.2)	1.00					
**Amount of cooking oil used for cooking**									
High	862 (33.8)	333 (31.3)	529 (35.6)	0.73	0.58–0.91	0.006*	0.78	0.62–0.99	0.037**
Moderate	1214 (47.6)	510 (47.9)	704 (47.3)	0.84	0.68–1.03	0.099	0.94	0.75–1.18	0.579
Low	476 (18.7)	221 (20.8)	255 (17.1)	1.00			1.00		
**BMI**									
Normal weight	1256 (42.2)	519 (48.8)	737 (49.5)	1.00					
Underweight	198 (7.8)	73 (6.9)	125 (8.4)	0.83	0.61–1.13	0.236			
Overweight	1098 (43.0)	472 (44.4)	626 (42.1)	1.07	0.91–1.26	0.414			
**Waist circumference**									
Normal	1290 (50.5)	500 (47.0)	790 (53.1)	1.00			1.00		
Obesity	1262 (49.5)	564 (53.0)	698 (46.9)	1.28	1.09–1.50	0.002*	1.30	1.09–1.54	0.003**

*Significance level at α = 0.05

**Significance level at α = 0.05 after controlling for sex, age, religion, education, occupation, income and marital status

One-half of the participants reported heavy use of salt for cooking (46.7%), heavy use of monosodium glutamate (50.9%), and heavy use of oil for cooking (33.8%). Almost half (43.0%) had a BMI defined as overweight, and 49.5% were defined as obese based on their waist circumference (Table 1).

Regarding triglyceride levels, 1064 participants (41.7%) were found to have abnormal serum triglycerides after being NPO for 8 h: 16.4% had a borderline high level of 150–199 mg/dL, and 25.3% had a high level of 200–499 mg/dL. One hundred eighty seven (17.6%) out of 1064 were diagnosed as HTG previously. Additionally, 107 out of 187 cases (57.2%) could control their serum triglyceride. The prevalence of abnormal HTG among males was 40.8, and 42.2% in females (Table 1).

In the univariate analysis, four variables were found to be associated with elevated serum triglycerides: tribe, religion, amount of oil used in daily cooking, and waist circumference. However, after controlling for sex, age, religion, education, occupation, income and marital status in the multivariate analysis, only three variables were found to be associated with elevated serum triglycerides: tribe, amount of cooking oil used daily and waist circumference. Members of the Lahu and Hmong tribes were 1.62 times (95% CI = 1.25–2.01) and 1.63 times (95% CI = 1.23–2.16) more likely to have elevated serum triglycerides than the members of the Akha tribe, respectively. Participants who used high quantities of cooking oil daily were 0.78 times (95% CI = 0.62–0.99) less likely to have triglyceride abnormalities than those who used low quantities of cooking oil, and those who had waist circumferences in the obese category were 1.30 times (95% CI = 1.09–1.54) more likely to have triglyceride abnormalities than those who had a normal waist circumference (Table 1).

In the correlation analysis between triglycerides and other key biomarkers, triglycerides were found to be significantly correlated with DBP (*r* = − 0.048, *p*-value = 0.013), HbA1c (*r* = 0.109, *p*-value< 0.001), HDL-C (*r* = − 0.436, *p*-value< 0.001), and LDL-C (*r* = − 0.099, *p*-value< 0.001). After classification by sex, the following four variables were found to have statistically significant correlations with triglycerides in males: SBP (*r* = − 0.090, *p*-value = 0.008), DBP (*r* = − 0.084, *p*-value = 0.013), HDL-C (*r* = − 0.042, *p*-value< 0.001), LDL-C (*r* = − 0.136, *p*-value< 0.001). Three variables were found to have statistically significant correlations with triglycerides in females: HbA1c (*r* = 0.138, *p*-value< 0.001), HDL-C (*r* = 0.444, *p*-value< 0.001), and LDL-C (*r* = − 0.081, *p*-value = 0.001) (Table 2).

**Table 2:** Correlations between triglycerides and key-biomarkers among the participants

Group	Correlation	Age	SBP	DBP	HbA1c	HDL-C	LDL-C
**Total**	***r***	−0.022	−0.016	−0.048	0.109	−0.436	−0.099
	***p-value***	*0.272*	*0.415*	0.013*	< 0.001*	< 0.001*	< 0.001*
**Male**	***r***	−0.062	−0.090	−0.084	0.047	−0.420	−0.136
	***p-value***	*0.068*	0.008*	0.013*	*0.168*	< 0.001*	< 0.001*
**Female**	***r***	−0.001	0.019	−0.036	0.138	0.444	−0.081
	***p-value***	*0.976*	*0.429*	*0.144*	< 0.001*	< 0.001*	0.001*

*SBP* Systolic blood pressure, *DBP* Diastolic blood pressure, *HDL-C* High density lipoprotein cholesterol, *LDL-C* Low density lipoprotein cholesterol, *HbA1c* Glycosylated hemoglobin

*Significant level at α = 0.05

## Discussion

The majority of hill tribe people aged 30 years and older in Thailand support their families using traditional agricultural crops, live in poor economic conditions, and have never been educated in the Thai educational system. They practiced unhealthy cooking in their daily life by using high amounts of salt, monosodium glutamate, and cooking oil. A large proportion of participants were overweight and got no regular exercise. The prevalence of elevated triglycerides in this study was 41.7%, and only 17.6% were diagnosed and treated properly. Members of the Lahu or Hmong tribes were more likely to have elevated triglycerides or HTG than those in the Akha tribe. Participants considered obese based on their waist circumference were also more likely to have elevated triglycerides than those persons of normal weight, although the participants who used high quantities of cooking oil were less likely to have elevated triglycerides than those who used a low quantity of cooking oil.

The prevalence of HTG in our study was 41.7%, which exceeds the overall rate from a study in Thailand that found 38.6% HTG [13]. While the American Association of Clinical Endocrinologists reported HTG in 39.0 and 27.8% of Mexican men and women, respectively, the rates were 27.8 and 22.7% among white American men and women, respectively [30], and 17.0% among people in Saudi Arabia [31]. However, a study in China found that the prevalence of HTG was 44.2%, which was similar to the rate found in the hill tribes in Thailand. This may be because most of the cooking practices and some of the cultural traditions are related to each other because the hill tribes in Thailand were originally from China [32].

It is clear that a large proportion of the hill tribe members aged 30 years and over in Thailand are living with HTG, which could possibly lead to the development severe diseases in the future. In addition, a large proportion of HTG patients did not receive proper care and treatment. This might be because the pathogenesis of HTG is not presented in severe forms, so most patients would not seek medical care. However, it should be investigated on the reason for a few proportions of the hill tribe HTG being treated and cared for. Furthermore, a community-based screening program could be one of the implementations to detect early stages of HTG and early treatment.

Even though there was no statistically significant association between education and HTG among the hill tribe people in the multivariate analysis, education could contribute to selected quality cooking oil to use in daily life. The hill tribe people could be improving their unhealthy behaviors if attending some level of education. Unfortunately, the majority of the hill tribe in Thailand did not attend a school (76.7%). A study in India was reported that those people who had a poor education were at a greater risk of having HTG than those who had high education [33].

In our study, we have found that some tribes (Lahu and Hmong tribes) had a higher risk of HTG than the others. One study performed in a large sample size from the United States in 2014 found that people aged 35 years and older with different races were found to have significantly different rates of HTG [34]. This finding was confirmed by a review in the United States that reported that participants of different races or ethnicities had different levels of HTG, even if they were living in very similar environment [35]. Therefore, differences in racial or ethnic backgrounds impact the level of triglyceride elevation.

In the present study, we found that participants who used high quantities of cooking oil tended have a lower triglyceride level. Additionally, hill tribe people today have totally changed their oil use from previous days (from animal oil to vegetable oil) [15, 16]. WHO [10] has reported that there are two sources of triglycerides: those consumed by eating and those produced by the human liver. However, the major source of triglycerides comes from the diet, particularly in meat and cooking oil [4]. Rygiel [6] reported that having a positive-energy balance diet with saturated fat and alcohol contributed to an increase in triglycerides, which was supported by a systemic review conducted in 2014 [36] that reported that a high fat diet and an inactive lifestyle were strongly associated with HTG. However, the difference in the direction of the association between the quantity of cooking oil used and the level of HTG could be related to the fact that most of the cooking oil used among hill tribe people has changed to vegetable oil. In one experimental study [37] that identified chemical compounds, many components of vegetable oils were found to be good for human health.

Additionally, it was found that in the overall analysis, triglycerides were positively correlated with DBP, HbA1C, and HDL-C, but they were negatively correlated with LDL-C. However, after classifying by sex, triglycerides in males were negatively correlated with SBP, DBP, HDL-C, and LDL-C, while triglycerides in females were found that be positively correlated with HbA1c and HDL-C and negatively correlated with LDL-C. The finding in females was supported by a longitudinal study in China [38], which found that triglycerides had a negative correlation with HDL-C and a marginal correlation with HbA1C. In our study, triglycerides were negatively correlated with DBP among males, which differed from a 12-year prospective cohort study among patients with diabetes conducted in Saudi Arabia [39] that found a positive correlation between triglycerides and blood pressure, although this difference may be due to the differences in the participants between the two studies. A study in Korea among people living in urban areas [40] found that triglycerides were correlated with HDL-C, which was similar to the findings in the current study.

Some limitations were found in the steps of the study. First, some older people could not provide information during data collection. However, the village health volunteer who was fluent in both Thai and their local languages was requested to help in improving the accuracy of the contents regarding the questions used. A few people (3 females) were not completely collected 5 mL blood specimens due to the difficulty to get into vein for the purpose (obesity). This might little interfere with the final interpretation of the findings, but it should be in the same direction of the associations found.

## Conclusion

Hill tribe people who live in northern Thailand live in poverty with low family incomes and poor education. Most health information and other key crucial media for health improvement in Thailand are produced in the Thai language. Therefore, due to the low level of education received within the Thai educational system among the hill tribe people, it is very difficult for them to access all of the important information from health care professionals to improve their health and the health of their family members. A large proportion of hill people were found to be overweight according to both BMI (60.7% were overweight) and waist circumference (49.5% were obese). Many use various substances, and relatively few people exercise regularly. Some tribes (Lahu and Hmong) were more likely to have elevated serum triglyceride levels than other tribes. Obesity is one of the risk factors for elevated serum triglycerides, although using a high quantity of cooking oil was found to serve as a protective factor for elevated serum triglycerides among hill tribe people aged 30 years and over. There is clearly a need for effective public health programs to be developed and implemented immediately to reduce the level of triglycerides by focusing on some of the tribes and on reducing body weight by encouraging regular exercise. Screening program to identify HTG among the risk population particularly who have high BMI should be considered, and those who have HTG problem should be cared and treated properly. Moreover, essential health information should be translated into the hill tribes’ local languages and distributed to improve accessibility for these people.

## Supplementary Information

**Additional file 1.** Questionnaire

**Additional file 2.**

## Data Availability

Raw data are available in attached file.

## Abbreviations

BMI: Body mass index
CVDs: Cardiovascular diseases
DBP: Diastolic blood pressure
HbA1c: Glycosylated hemoglobin
HDL-C: High density lipoprotein cholesterol
HTG: Hypertriglyceridemia
IQR: Interquartile range
LDL-C: Low density lipoprotein cholesterol
NPO: Nothing per oral
SBP: Systolic blood pressure
SD: Standard deviation
WHO: World Health Organization

## References

[1] Jacobson TA, Ito MK, Maki KC, Orringer CE, Bays HE, Jones PH, et al. National lipid association recommendations for patients-centered management of dyslipidemia: part-II-full report. J Clin Lipidol. 2015;9:S1-S122. 10.1016/j.jacl.2015.02.003 25911072

[2] World Health Organization (WHO). Cardiovascular diseases. Available from: https://www.who.int/news-room/fact-sheets/detail/cardiovascular-diseases-(cvds). Accessed 16 June 2020.

[3] Brahm A, Hegele RA (2013). Hypertriglyceridemia. Nutrients.

[4] American College of Cardiology (ACC). Hypertriglyceridemia management according to the 2018 AHA/ACC guideline. Available from: https://www.acc.org/latest-in-cardiology/articles/2019/01/11/07/39/hypertriglyceridemia-management-according-to-the-2018-aha-acc-guideline. Accessed 16 June 2020.

[5] American Family Physician. Practice guidelines: endocrine society releases guidelines on diagnosis and management of hypertriglyceridemia. Available from: https://www.aafp.org/afp/2013/0715/p142.pdf. Accessed 16 June 2020. 23939649

[6] Rygiel K (2018). Hypertriglyceridemia: common causes, prevention and treatment. Curr Cardiol Rev.

[7] Antonios N, Angiolillo DJSS (2008). Hypertriglyceridemia and ischemic stroke. Eur Neurol.

[8] Pretis ND, Amodio A, Frulloni L (2018). Hypertriglyceridemic pancreatitis: epidemiology, pathophysiology and clinical management. United European Gastroenterol J.

[9] Peng J, Luo F, Ruan G, Peng R, Li X. Hypertriglyceridemia and atherosclerosis. Lipids Health Dis. 2017;16(233). 10.1186/s12944-017-0625-0. 10.1186/s12944-017-0625-0 PMC5719571 29212549

[10] World Health Organization (WHO). Obesity and overweight. https://www.who.int/en/news-room/fact-sheets/detail/obesity-and-overweight. Accessed 16 June 2020.

[11] Laufs U, Parhofer KG, Ginsberg HN, Hegele RA (2020). Clinical review on triglycerides. Eur Heart J.

[12] Centers for Disease Control and Prevention (CDC). National center for health statistics: trends in elevated triglyceride in adults: United States, 2001-2012. Available from: https://www.cdc.gov/nchs/products/databriefs/db198.htm. Accessed 18 June 2020.

[13] Aekplakorn W, Taneepanichskul S, Kessomboon P, Chongsuvivatwong V, Putwatana P, Sritara P, Sangwatanaroj S, Chariyalertsak S (2014). Prevalence of dyslipidemia and management in the Thai population, National Health Examination Survey IV, 2009. J Lipids.

[14] United Nations (UN). Thailand: country profile. Available from: http://data.un.org/CountryProfile.aspx/_Images/CountryProfile.aspx?crName=Thailand. Accessed 18 June 2020.

[15] Watanasin R (2012). Thai food: a gateway to cultural understanding. J Royal Inst Thailand.

[16] Pragattakomol P (2018). The evolution of cultural landscape and built environment through Thai food and way of living: the case study of central region of Thailand. Dusit Thani Coll J.

[17] The World Bank. Thailand economic monitor reports. Available from: https://www.worldbank.org/en/country/thailand/publication/thailand-economic-monitor-reports. Accessed 21 June 2020.

[18] International Organization for Migration (IOM). Thailand migration report 2019. Available from: https://thailand.iom.int/sites/default/files/document/publications/Thailand%20Report%202019_22012019_HiRes.pdf. Assessed 5 July 2020.

[19] Princess Maha Chakri Siridhorn Anthropology center. Hill tribe. 2017. Available from: http://www.sac.or.th/main/index.php. Assessed 5 July 2020.

[20] Apidechkul T, Wongnuch PW, Sittisarn S, Ruanjai T (2016). Health status of Akha hill tribe in Chiang Rai Province, Thailand. J Pub Health Dev.

[21] Tamornpark R, Apidechkul T, Upala P, Inta C (2017). Factors associated with type 2 diabetes mellitus among the elderly hill tribe population in Thailand. Southeast Asian J Trop Med Public Health.

[22] Apidechkul T (2018). Prevalence and factors associated with type 2 diabetes mellitus and hypertension among the hill tribe elderly population in northern Thailand. BMC Public Health.

[23] Apidechkul T, Laingoen O, Suwannaporn S (2016). Inequity in accessing health care service in Thailand in 2015: a case study of the hill tribe people in Mae Fah Luang District, Chiang Rai, Thailand. J Health Res.

[24] The Hill tribe Welfare and Development Center, Ministry of Interior (2017). The hill tribe population in Thailand.

[25] Department of Mental Health. Stress test-5 (ST-5). Available from: https://www.dmh.go.th/test/download/view.asp?id=18. Assessed 11 July 2020.

[26] Kroenke K, Spitzer R, Williams JB (2001). The PHQ-9 validity of a brief depression severity measure. J Gen Intern Med.

[27] World Health Organization (WHO). Body mass index-BMI. Available from: http://www.euro.who.int/en/health-topics/disease-prevention/nutrition/a-healthy-lifestyle/body-mass-index-bmi. Accessed 11 July 2020.

[28] World Health Organization (WHO). Waist circumference and waist-hip ratio: report of a WHO expert consultation. Available from: https://www.who.int/publications/i/item/9789241501491. Accessed 14 July 2020.

[29] World Health Organization (WHO). Guideline for the management of dyslipidemia in patients with diabetes mellitus: quick reference guide. Available from: https://apps.who.int/iris/handle/10665/119809. Accessed 14 July 2020.

[30] Berglund L, Brunzell JD, Goldberg AC, Goldberg IJ, Sacks F, Murad MH, et al. Evaluation and treatment of hypertriglyceridemia: an endocrine society clinical practice guideline. J Clin Endocrinol Metab. 2012;97(9):2969-89. 10.1210/jc.2011-3213 PMC3431581 22962670

[31] Al-Hassan YT, Fabella EL, Estrella E, Aatif M (2018). Prevalence and determinants of dyslipidemia: data from a Saudi University clinic. Open Public Health J.

[32] Qi L, Ding X, Tang W, Li Q, Mao D, Wang Y (2015). Prevalence and risk factors associated with dyslipidemia in Chongqing, China. Int J Environ Res Public Health.

[33] Gupta R, Deedwania PC, Sharma K, Gupta A, Guptha S, Achari V (2012). Association of education, occupational and socioeconomic status with cardiovascular risk factors in Asian Indians: a cross-sectional study. PlosOne.

[34] Frank AT, Zhao B, Jose PO, Azar KM, Fortman SP, Palaniappan LP (2014). Racial/ethnic differences in dyslipidemia patterns. Circulation..

[35] Morris A, Ferdinand KC (2009). Hyperlipidemia in racial/ethnic minorities: differences in lipid profiles and the impact of statin therapy. Clin Lipid.

[36] Tenenbaum A, Klempfner R, Fisman EZ (2014). Hypertriglyceridemia: a tool long unfairly neglected major cardiovascular risk factor. Cardiovasc Diabetol.

[37] Fakhri NA, Qadir HK (2011). Separation, identification and determination of triglycerides in vegetable oil samples. J Food Sci Eng.

[38] Tao LX, Liu XT, Zhu HP, Luo YX, Guo J, Wu LJ (2016). Longitudinal associations between triglycerides and metabolic syndrome components in a Beijing adult population, 2007-2012. Int J Med Sci.

[39] Aziz KM (2017). Association of serum lipids with high blood pressure and hypertension among diabetes patients: mathematical regression models to predict blood pressure from lipids, an experience from 12 years follow up of more than 9,000 patients’ cohort. Gen Med.

[40] Park HR, Shin SR, Han A, Jeong YJ (2015). The correlation between the triglycerides to high density lipoprotein cholesterol ratio and computed tomography-measured visceral fat and cardiovascular disease risk factors in local adult male subjects. Korean J Fam Med.

