# Supplementary material for: Factors associated with hypertriglyceridemia among the hill tribe people aged 30 years and over, Thailand: a cross-sectional study
Source: BMC Public Health. 2021 Mar 23;21:581. doi: 10.1186/s12889-021-10632-z (PMC7989000; doi:10.1186/s12889-021-10632-z)
Supplement: Supplementary file 1 — Additional file 1. Questionnaire [file 12889_2021_10632_MOESM1_ESM.pdf]

Question guideline

1. How do you cook for daily food?
2. Anyone of your family member use substance?
3. Can you please explain the daily life activities to those who are aged 30 years and over in your family?
4. Can you explain the problem of human body lipid?
5. Do you have any problem in your life? what and how?
6. How do you receive health information?

Questionnaire of the project of assessing abnormal triglycerides

Part I general information

1. Sex

    Male   Female

2. Age……………………Year
3. Education
    - No-education
    - Primary school
    - High school
    - University degree
4. Tribe
    - Akha
    - Lahu
    - Hmong
    - Yao
    - Karen
    - Lisu
5. Religion
    - Buddhist
    - Christian
    - Other
6. Occupation
    - Unemployed
    - Agriculturalist
    - Trader or other
7. Annual income…………………….baht
8. Marital status
    - Single
    - Married
    - Ever married
9. Having debt
    - Yes
    - No

Part II Health behaviors and substance use

1. Exercise
    - No

Sometimes
Regular

2. Amount of salt use for cooking
   Low
   Moderate
   High
3. Amount of monosodium glutamate use for cooking
   Low
   Moderate
   High
4. Amount of cooking oil used for cooking
   Low
   Moderate
   High

5.Alcohol use
   Yes
   No
6. Smoking
   Yes
   No
7. Amphetamine use
   Yes
   No
8. Opium use
   Yes
   No
9. Glue use
   Yes
   No
10. Marijuana use
   Yes
   No

Part III ST-5 and PHQ-9

The questions asking stress and depression please follow the original form in references no.26, and 27, respectively.

Part IV Physical examination

Weight………………………………………………....kg
Height……………………………………..………cm
Waist circumference …………………………………..cm
LDL ……………………………………………... mg/dL
HDL………………………………………………...mg/DL
Triglycerides……………………………………..mg/DL
HbA1c……………………………………………..
